# Cyclic Glucans Enhance Solubility of Bioavailable Flavonoids

**DOI:** 10.3390/molecules21111556

**Published:** 2016-11-16

**Authors:** Seyeon Park

**Affiliations:** Department of Applied Chemistry, Dongduk Women’s University, Seoul 136-714, Korea; sypark21@dongduk.ac.kr; Tel.: +82-2-940-4514; Fax: +82-2-940-4510

**Keywords:** β-cyclodextrin, cyclosophoraoses, flavonoid, inclusion complex, bioavailability

## Abstract

Diverse flavonoids are abundant in dietary food constituents and possess useful biological activities. However, some flavonoids have limited bioavailability due to their low solubility in water. As an important approach to enhance aqueous solubility, inclusion of hydrophobic guest molecules in hydrophilic hosts such as cyclic glucans has been used. This review summarizes applications of β-cyclodextrin, synthetic β-cyclodextrin derivatives, and newly synthesized derivatives of cyclosophoraoses as complexing agents to enhance the bioavailability of flavonoids such as baicalein, kaempferol, and naphthoflavones.

## 1. Introduction

**Figure 1:**
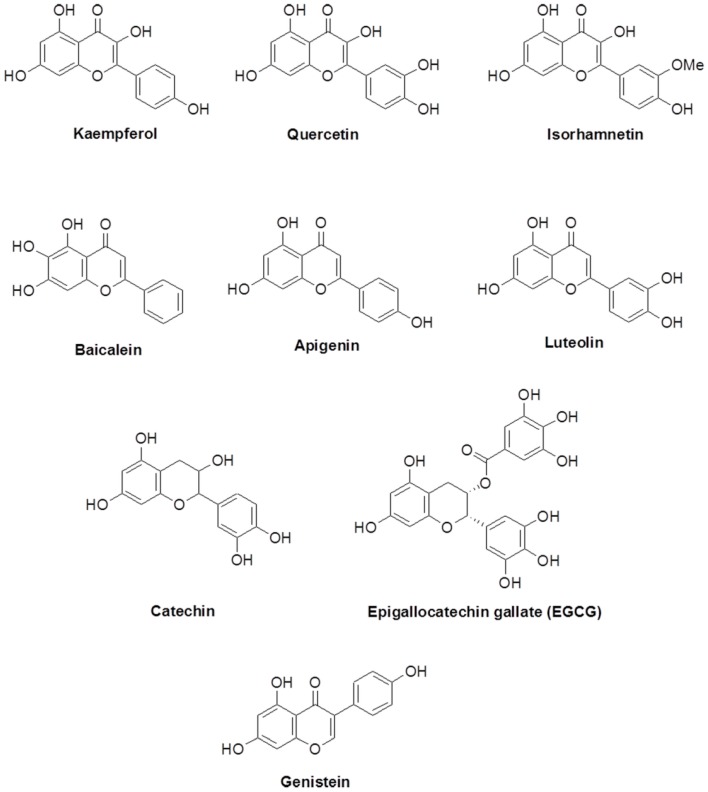
Structure of flavonoids.

Flavonoids are polyphenols that are abundant in dietary food constituents. They are abundant in numerous groups of natural products such as fruits, vegetables, grains, and nuts. Flavonoids have many roles associated with disease curing and intracellular signaling. These roles have been reviewed previously [1,2]. Flavonoids possess a common phenylbenzopyrone structure (C6-C3-C6). They can be categorized as flavanol, flavone, flavanone, and isoflavone according to the substituted group and direction of the phenyl ring as shown in Figure 1. Kaempferol, quercetin, and isorhamnentin are categorized as flavonols. Baicalein, apigenin, and luteolin are categorized as flavones. Catechin and epigallocatechin are flavanols. Genistein is an isoflavone.

Flavonoids possess a variety of biological activities at non-toxic concentrations in organisms [2]. The role of dietary flavonoids and the underlying mechanisms in cancer prevention have been widely suggested. Flavonoids may affect tumorigenesis [3] with anti-oxidant activities [4] by scavenging free radical mutagens and carcinogens [5]. Flavonoids also contribute to UV protection. They can modulate enzymatic activity and offer protection against viral, fungal, and bacterial infections [1]. In addition, a variety of flavonoids can directly interact with intracellular proteins and affect intracellular signaling [2]. Some dietary flavonoids are known to be transformed to phytoestrogen by gut microflora [1].

However, some flavonoid compounds have little biological activities in vivo when little or none of the compound gets solubilized in the cytoplasmic environment [6]. Consequently, knowing how much of a flavonoid nutrient is present in specific food or dietary supplement and knowing how much of it is bioavailable are equally important.

Recently, several cyclic glucans have received attention because of their potential as carriers. These compounds consist of glucose monomers arranged in a toroidal ring. They are formed during bacterial digestion of cellulose.

Cyclodextrins (CDs) are a family of macrocyclic oligosaccharides known as α, β, and γ-CD (composed of 6-, 7-, and 8-α-(1,4) linked glycosyl units, respectively). These compounds have a characteristic cone-shaped and internally truncated cavity. Compound β-CD is an α-1,4-linked macrocyclic oligosaccharide containing seven glucose units. It can encapsulate hydrophobic compounds in its internal cavity [7]. However, torus-shaped hydrophobic cavity inside of β-CD with a diameter of 6.0–6.5 Å and a height of 7.9 Å is relatively small for some target guest compounds such as β-naphthoflavone. Therefore, 6,6′-thiobis(methylene)-β-cyclodextrin dimer has been synthesized and used as a host [8].

Cyclosophoraoses (Cys) are cyclic β-(1,2)-glucans containing 10–40 glucose residues. They are produced from the metabolic process of microorganism belonging to genus *Agrobacterium* within family of *Rhizobiaceae* [9]. Cys has been known to form inclusion complexes with a variety of hydrophobic guest molecules and enhance their water solubilities [10]. Cys derivatives could be synthesized by substitution of various functional groups such as methyl, hydroxypropyl, carboxymethyl, and sulfate groups. They have been used as hosts of inclusion complex [11]. In addition, Cys and Cys derivative have chiral selectivity on enantiomers [12,13,14,15].

**Figure 2:**
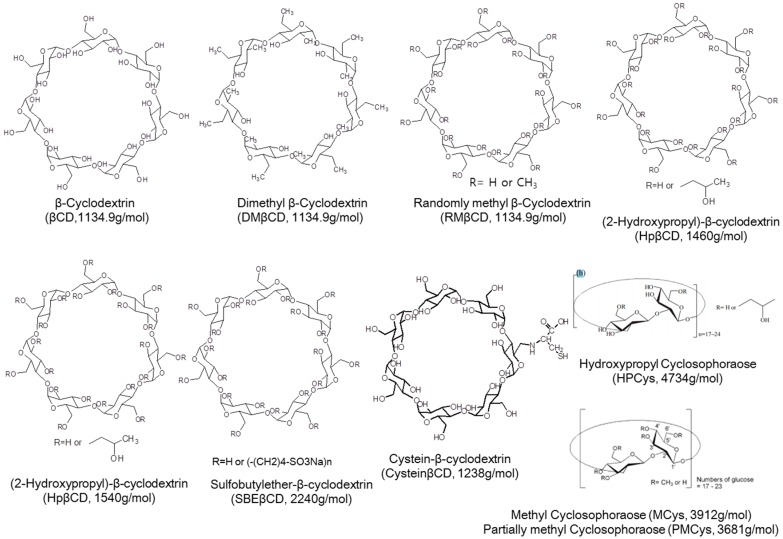
A variety of host molecules.

This paper reviewed the enhanced solubility of flavonoids in complex with cyclic glucans including CD, Cys, and newly synthesized cyclic glucans (Figure 2).

## 2. Cysteinyl β-Cyclodextrin-Baicalein Inclusion Complex

Many studies have shown that baicalein exerts anticancer and anti-inflammation activities [16]. Baicalein can induce apoptosis and cell cycle arrest, leading to inhibition of cell proliferation of human prostate, hepatocellular, breast, and myeloma cancer cells [17,18]. Moreover, baicalein can suppress the migration and invasion of human skin, hepatoma, and breast cancer cells [19,20,21]. Baicalein also suppresses the release of prostaglandin E2 (PGE2) and arachidonic acid (AA) by inhibiting MAPK-cytosolic phospholipase A2 (cPLA2) pathway, leading to anti-inflammatory effects [22].

**Figure 3:**
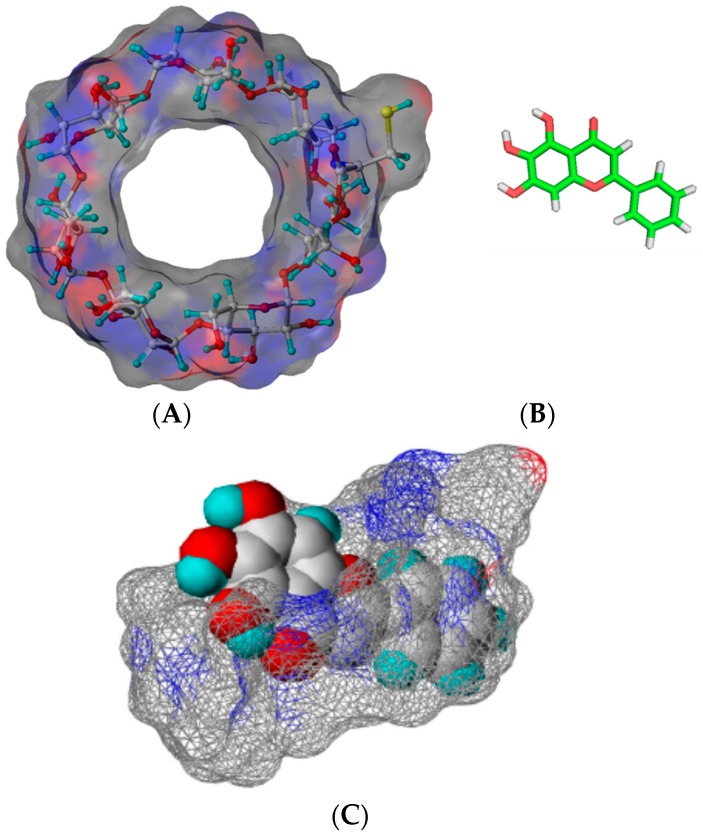
Structures of cysteinyl β-CD (**A**) and baicalein (**B**). The proposed mode of complex (**C**) [24].

Inclusion complexes formed between baicalein and different kinds of cyclodextrins have been reported [23,24]. The solubility of baicalein in the presence of natural (α-, β-, and γ-) cyclodextrins and its derivatives including hydroxypropyl-β-cyclodextrin (HP-β-CD), (2,6-di-*O*-methyl)-β-cyclodextrin (DM-β-CD), and cysteinyl-β-cyclodextrin (cysteinyl-β-CD) is higher than that of free baicalein. When the solubilizing efficiency is determined as the ratio between the solubility of the drug in the presence of a certain host concentration and the baicalein alone in water, the solubilizing efficiencies of HP-β-CD and DM-β-CD are about four and five times of β-CD in the presence of 10 mmol of host molecules [23]. Recently, Jung et al. have reported that the aqueous solubility of β-CD could be improved about 100-fold (cysteinyl β-CD: >180 g/100 mL, 25 °C vs. β-CD: 1.82 g/100 mL, 25 °C) after attaching cysteine to β-CD [24]. Therefore, the solubilizing efficiency of cysteinyl β-CD is about four times that of the original β-CD in the presence of 10 mM β-CDs [24]. The solubility of baicalein using cysteinyl β-CD (~5 mM) was almost twice of that of DM-β-CD (~3 mM) at a saturated condition. Regarding the mode of inclusion complexation, shifts of proton signals of baicalein in the presence of DM-β-CD have indicated that B-ring, C-ring, and A-ring are included in the cavity of DM-β-CD [23]. The structures of cysteinyl β-CD and baicalein are shown in Figure 3. The complexation with cysteinyl β-CD has resulted in more effective antioxidant activity of baicalein [23].

## 3. Inclusion Complexes of α- and β-Naphthoflavone

α- and β-naphthoflavone (7,8-benzoflavone and 5,6-benzoflavone, respectively) are synthetic flavonoids. Their difference is in the direction of the flavone naphthyl ring. They have negligible aqueous solubility because of their extremely low hydroxylation. The bioavailability of these naphthoflavones has been suggested as enzyme regulators [8,25]. Especially, α-naphthoflavone can modulate xenobiotic metabolism as an inhibitor of aryl hydrocarbon receptor (AhR), a ligand-activated transcription factor that promotes the expression of cytochrome P450 (CYP) isoforms CYP1A1 and CYP1A2 [26,27,28]. In addition, α-naphthoflavone is inhibitors of CYP19 (aromatase), CYP1A1, CYP1A2, and CYP1B1 (IC_50_s = 500, 60, 6, and 5 nM, respectively) [29,30,31].

It has been reported that β-naphthoflavone is a putative chemopreventive agent as a potent agonist of the AhR. Therefore, it is an inducer of detoxification enzymes such as cytochromes P450 (CYPs) and uridine 5′-diphospho-glucuronosyltransferases (UGTs) [32,33]. Our previous study has shown that β-naphthoflavones (5,6-benzoflavone series) has inhibitory activity on β-catenin/Tcf signaling by directly blocking complex formation of β-catenin/Tcf with DNA. Activated β-catenin signaling is related to the oncogenic transformation of certain cancer cells [34].

**Figure 4:**
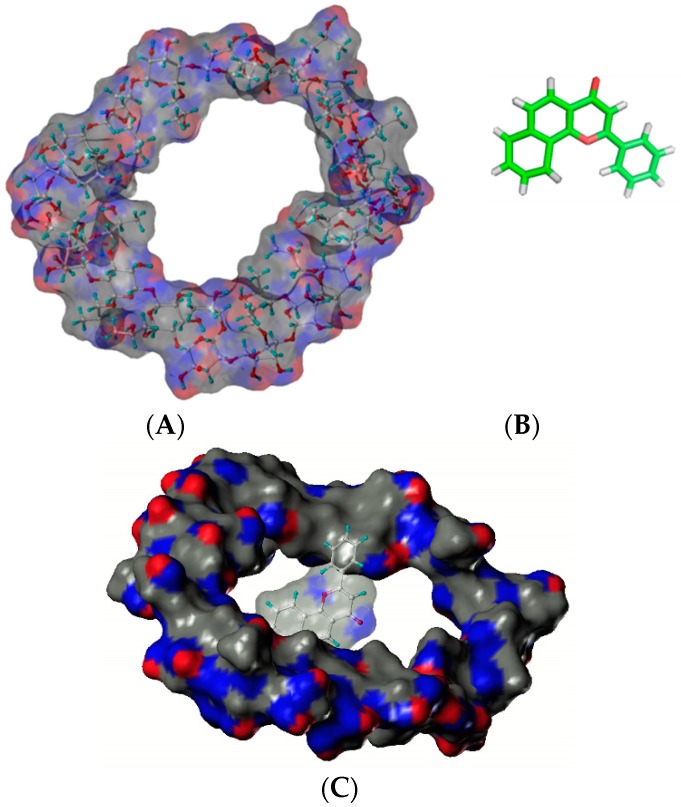
Structures of HP-Cys (**A**) and α-naphthoflavone (**B**). The proposed mode of complex (**C**) [25].

Hydroxypropyl cyclosophoraoses (HP-Cys), a Cys derivative synthesized by substitution of the hydroxypropyl group, can enhance the aqueous solubility of α-naphthoflavone as a guest up to 257-fold after the addition of 8 mM HP Cys (Figure 4) [25]. NMR and molecular modeling results have suggested that HP Cys can form complexes with α-naphthoflavone by accommodating the three hydrophobic hydroxypropyl groups on top of sugar rings [25]. Structures of HP-Cys and α-naphthoflavone are shown in Figure 4.

**Figure 5:**
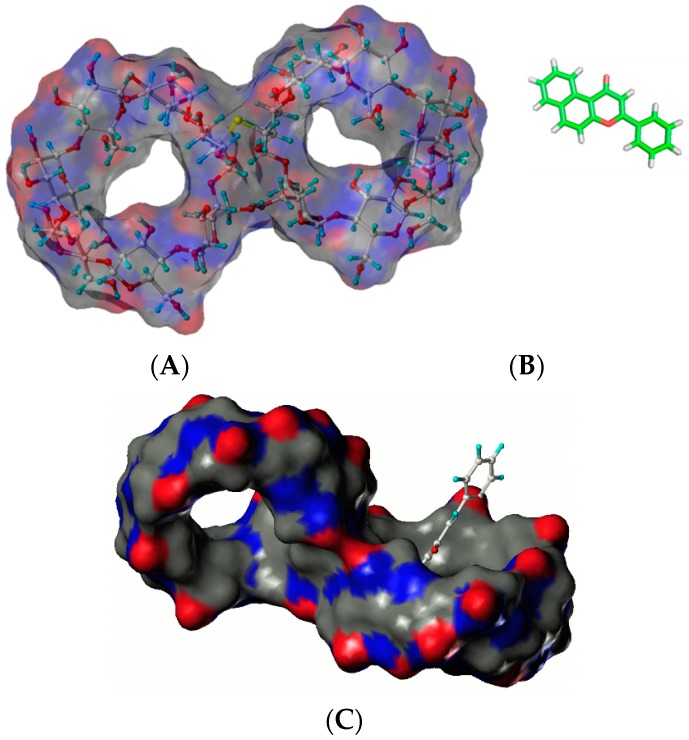
Structures of β-CD dimer (**A**) and β-naphthoflavone (**B**). The proposed mode of complex (**C**) [8].

When β-naphthoflavone is used in an inclusion complex with newly synthetic 6,6′-thiobis(methylene)-β-cyclodextrin dimer, the solubility of the β-naphthoflavone is enhanced [8]. Structures of β-CD dimer β-naphthoflavone are shown in Figure 5. Formation of an inclusion complex of β-CD dimer can increase the solubilization of β-naphthoflavone up to 469-fold and enhance its bioavailability as an AhR agonist.

## 4. Flavonol and Glucans

Dimeric β-cyclodextrin linked by a thioether bridge can enhance the solubility of β-naphthoflavone and flavonol such as myricetin, quercetin, and kaempferol [35]. The aqueous solubility of myricetin, quercetin, and kaempferol is enhanced 33.6-, 12.4-, and 10.5-fold after complex formation with 9 mM dimeric β-CD [35].

Quercetin has been reported to have beneficial effects on human health. Quercetin has shown an anti-asthmatic effect and anti-inflammatory effects in a murine model [36,37,38,39]. In vitro quercetin also has an anti-allergy effect in mast cells and basophils by inhibiting histamine release [40,41]. Several in vitro and in vivo studies have indicated that quercetin has anticancer, antioxidant, antiproliferative, and proapoptotic effects. It has a synergistic effect with some chemotherapeutic agents [42,43,44,45,46].

Kaempferol is also abundant in fruits and vegetables such as tea, broccoli, apples, strawberries, and beans [47]. A variety of in vitro studies have indicated that kaempferol can induce apoptosis in cancer cells by modulating the MAPK pathway [47,48,49].

In vitro studies have suggested that kaempferol can inhibit the expression levels of both interleukin-1β and tumor necrosis factor, inflammatory response inducing cytokines [50]. It has been demonstrated that kaempferol can disrupt NF-κB, thereby preventing induction of its gene targets [47,51]. Furthermore, quercetin and kaempferol can inhibit the direct binding of β-catenin/Tcf with DNA, leading to suppression of wnt/β-catenin signaling implicated in human carcinogenesis [2].

Generally, the restricted bioavailabilities of kaempferol and quercetin are due to their poor dissolutions in a number of solvents, including water. Kaempferol is poorly absorbed into the bloodstream. It cannot force its way into cells to manipulate signaling pathways or inhibit certain protein functions [47]. In studies on the bioavailability of quercetin as a dietary supplement, quercetin from different dietary sources has been found to be more bioavailable when a sugar is attached to quercetin [52]. Quercetin glycosides are better absorbed because of their improved solubilities [52]. Taken together, enhancing the solubility of flavonoids formed by inclusion complexation with cyclic glucans is essential to enhance their bioavailabilities.

**Figure 6:**
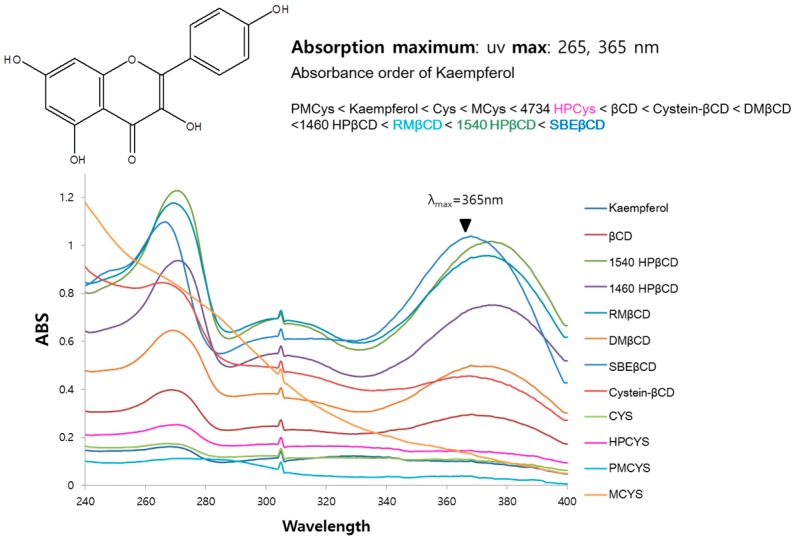
UV absorption spectra of kaempferol with or without 1 mM of host molecules in water.

To screen effective host molecules of kaempferol, inclusion complexes with a variety of cyclic glucans were prepared using a suspension method (cyclic glucans were distributed from the Microbial Carbohydrate Resource Bank (MCRB) at Konkuk University, Seoul, Korea). Briefly, kaempferol was dissolved in methanol and then added to an aqueous solution of an appropriate concentration of glucans. The resulting mixture was equilibrated and lyophilized. The amount of dissolved kaempferol was analyzed using UV-Vis spectrophotometry. The spectra were obtained in the range 240–400 nm as shown in Figure 6. Solubility enhancement of guest kaempferol was maximized by sulfobutylether-β-CD. Therefore, this compound is considered as the most suitable host.

## 5. Practical Application

Changing guest molecular properties after formation of inclusion complexes has led to many applications of cyclic glucans in pharmaceuticals, cosmetics, and food industries [53]. As a practical application of cyclic glucans in the cosmetic industry, the formation of an inclusion complex between cyclodextrin and a variety of organic compounds can increase the stability and solubility of cosmetic active ingredients and protect them from light- and oxidation-induced degradation [53,54]. Moreover, complexation of a drug with cyclodextrin might reduce problems associated with topical formulation such as poor aqueous solubility, incorporation of vehicle, photodegradation, and skin irritation [55]. Cyclodextrins have also been used in systems to reduce percutaneous absorption of common sunscreen agents [56,57]. Considering these practical applications of pre-existing cyclodextrin, more efficient and newly synthesized cyclic glucans could expand their applications in different industries.

## 6. Conclusions

In this paper, recent advances in supramolecular assembly of flavonoids with synthesized β-CD or Cys derivatives are reviewed. Synthesized β-CD or Cys derivatives are able to promote the aqueous solubility of flavonoids as novel host molecules. In addition, different kinds of Cys derivatives have specificity for their guest molecules because of their variations in internal cavity size and charges formed by different modifications. When these flavonoids are used in supramolecular complexes, their biological activities are enhanced, thereby promoting bioavailability. Based on this review, a variety of synthesized β-CD or Cys derivatives could broaden the range of applications of cycloglucans as a solubilizing adjuvant for medicines and cosmetics.

## References

[1] Andersen Ø.M., Markham K.R. (2006). FLAVONOIDS Chemistry, Biochemistry and Applications.

[2] Park S. (2015). Polyphenol Compound as a Transcription Factor Inhibitor. Nutrients.

[3] Kuntz S., Wenzel U., Daniel H. (1999). Comparative analysis of the effects of flavonoids on proliferation, cytotoxicity, and apoptosis in human colon cancer cell lines. Eur. J. Nutr..

[4] Böhm H., Boeing H., Hempel J., Raab B., Kroke A. (1998). Flavonols, flavone and anthocyanins as natural antioxidants of food and their possible role in the prevention of chronic diseases. Ernahrungswiss.

[5] Williamson G., Faulkner K., Plumb G.W. (1998). Glucosinolates and phenolics as antioxidants from plant foods. Eur. J. Cancer Prev..

[6] D’Archivio M., Filesi C., Varì R., Scazzocchio B., Masella R. (2010). Bioavailability of the polyphenols: Status and controversies. Int. J. Mol. Sci..

[7] Crini G. (2014). Review: A history of cyclodextrins. Chem. Rev..

[8] Choi J.M., Cho E., Lee B., Jeong D., Choi Y., Yu J.H., Jung S. (2016). Enhancing bio-availability of β-naphthoflavone by supramolecular complexation with 6,6′-thiobis(methylene)-β-cyclodextrin dimer. Carbohydr. Polym..

[9] Breedveld M.W., Miller K.J. (1994). Cyclic β-glucans of members of the family Rhizobiaceae. Microbiol. Rev..

[10] Koizumi K., Okada Y., Horiyama S., Utamura T., Higashiura T., Ikeda M. (1984). Preparation of cyclosophoraose-A and its complex-forming ability. J. Incl. Phenom..

[11] Cho E., Jung S. (2015). Supramolecular Complexation of Carbohydrates for the Bioavailability Enhancement of Poorly Soluble Drugs. Molecules.

[12] Jeong D., Kim H., Dindulkar S.D., Lee J.Y., Jung S. (2015). Preparation of a Novel Chiral Stationary Phase Containing Rhizobial Cyclic β-(1→2) Glucans for the Chiral Separation of Some Flavonoids. Bull. Korean Chem. Soc..

[13] Jeon Y., Kwon C., Cho E., Jung S. (2010). Carboxymethylated cyclosophoraose as a novel chiral additive for the stereoisomeric separation of some flavonoids by capillary electrophoresis. Carbohydr. Res..

[14] Lee S., Choi Y., Lee S., Jeong K., Jung S. (2004). Chiral recognition based on enantioselective interactions of propranolol enantiomers with cyclosophoraoses isolated from *Rhizobium meliloti*. Chirality.

[15] Lee S., Jung S. (2003). Enantioseparation using cyclosophoraoses as a novel chiral additive in capillary electrophoresis. Carbohydr. Res..

[16] Ying G., Snyder S.A., Smith J.N., Chen Y.C. (2016). Anticancer properties of baicalein: A review. Med. Chem. Res..

[17] Chen H., Gao Y., Wu J., Chen Y., Chen B., Hu J., Zhou J. (2014). Exploring therapeutic potentials of baicalin and its aglycone baicalein for hematological malignancies. Cancer Lett..

[18] Li H.L., Zhang S., Wang Y., Liang R.R., Li J., An P., Wang Z.M., Yang J., Li Z.F. (2013). Baicalein induces apoptosis via a mitochondrial-dependent caspase activation pathway in T24 bladder cancer cells. Mol. Med. Rep..

[19] Chiu Y.W., Lin T.H., Huang W.S., Teng C.Y., Liou Y.S., Kuo W.H., Lin W.L., Huang H.I., Tung J.N., Huang C.Y. (2011). Baicalein inhibits the migration and invasive properties of human hepatoma cells. Toxicol. Appl. Pharm..

[20] Wang L., Ling Y., Chen Y., Li C.L., Feng F., You Q.D., Lu N., Guo Q.L. (2010). Flavonoid baicalein suppresses adhesion, migration and invasion of MDA-MB-231 human breast cancer cells. Cancer Lett..

[21] Wu B., Li J., Huang D., Wang W., Chen Y., Liao Y., Tang X., Xie H., Tang F. (2011). Baicalein mediates inhibition of migration and invasiveness of skin carcinoma through Ezrin in A431 cells. BMC Cancer.

[22] Nakahata N., Kutsuwa M., Kyo R., Kubo M., Hayashi K., Ohizumi Y. (1998). Analysis of inhibitory effects of scutellariae radix and baicalein on prostaglandin E2 production in rat C6 glioma cells. Am. J. Chin. Med..

[23] Zhou Q., Wei X., Dou W., Chou G., Wang Z. (2013). Preparation and characterization of inclusion complexes formed between baicalein and cyclodextrins. Carbohydr. Polym..

[24] Kim H., Yiluo H., Park S., Lee J.Y., Cho E., Jung S. (2016). Characterization and Enhanced Antioxidant Activity of the Cysteinyl β-Cyclodextrin-Baicalein Inclusion Complex. Molecules.

[25] Piao J., Jang A., Choi Y., Tahir M.N., Kim Y., Park S., Cho E., Jung S. (2014). Solubility enhancement of α-naphthoflavone by synthesized hydroxypropyl cyclic-(1→2)-β-d-glucans (cyclosophoroases). Carbohydr. Polym..

[26] Denison M.S., Nagy S.R. (2003). Activation of the aryl hydrocarbon receptor by structurally diverse exogenous and endogenous chemicals. Annu. Rev. Pharmacol. Toxicol..

[27] Smith K.J., Murray I.A., Tanos R., Tellew J., Boitano A.E., Bisson W.H., Kolluri S.K., Cooke M.P., Perdew G.H. (2011). Identification of a high-affinity ligand that exhibits complete aryl hydrocarbon receptor antagonism. J. Pharmacol. Exp. Ther..

[28] Wilhelmsson A., Whitelaw M.L., Gustafsson J.A., Poellinger L. (1994). Agonistic and antagonistic effects of alpha-naphthoflavone on dioxin receptor function. Role of the basic region helix-loop-helix dioxin receptor partner factor Arnt. J. Biol. Chem..

[29] Bruno R.D., Njar V.C. (2007). Targeting cytochrome P450 enzymes: A new approach in anti-cancer drug development. Bioorg. Med. Chem..

[30] Campbell D.R., Kurzer M.S. (1993). Flavonoid inhibition of aromatase enzyme activity in human preadipocytes. J. Steroid Biochem. Mol. Biol..

[31] Sineva E.V., Rumfeldt J.A., Halpert J.R., Davydov D.R. (2013). A large-scale allosteric transition in cytochrome P450 3A4 revealed by luminescence resonance energy transfer (LRET). PLoS ONE.

[32] Chlouchi A., Girard C., Bonet A., Viollon-Abadie C., Heyd B., Mantion G., Martin H., Richert L. (2007). Effect of chrysin and natural coumarins on UGT1A1 and 1A6 activities in rat and human hepatocytes in primary culture. Planta Med..

[33] Izzotti A., Bagnasco M., Cartiglia C., Longobardi M., Camoirano A., Tampa E., Lubet R.A., De Flora S. (2005). Modulation of multigene expression and proteome profiles by chemopreventive agents. Mutat. Res..

[34] Park S., Choi J. (2010). Inhibition of β-catenin/Tcf signaling by flavonoids. J. Cell. Biochem..

[35] Cho E., Jeong D., Paik H., Jung S. (2014). Solubility Enhancement of Flavonols in the Inclusion Complex with Thioether-bridged Dimeric β-Cyclodextrins. Bull. Korean Chem. Soc..

[36] Joskova M., Franova S., Sadlonova V. (2011). Acute bronchodilator effect of quercetin in experimental allergic asthma. Bratisl. Lek. Listy.

[37] Jung C.H., Lee J.Y., Cho C.H., Kim C.J. (2007). Anti-asthmatic action of quercetin and rutin in conscious guinea-pigs challenged with aerosolized ovalbumin. Arch. Pharm. Res..

[38] Moon H., Choi H.H., Lee J.Y., Moon H.J., Sim S.S., Kim C.J. (2008). Quercetin inhalation inhibits the asthmatic responses by exposure to aerosolized-ovalbumin in conscious guinea-pigs. Arch. Pharm. Res..

[39] Rogerio A.P., Dora C.L., Andrade E.L., Chaves J.S., Silva L.F., Lemos-Senna E., Calixto J.B. (2010). Anti-inflammatory effect of quercetinloaded microemulsion in the airways allergic inflammatory model in mice. Pharmacol. Res..

[40] Bronner C., Landry Y. (1985). Kinetics of the inhibitory effect of flavonoids on histamine secretion from mast cells. Agents Actions.

[41] Fox C.C., Wolf E.J., Kagey-Sobotka A., Lichtenstein L.M. (1988). Comparison of human lung and intestinal mast cells. J. Allergy Clin. Immunol..

[42] Braganhol E., Zamin L.L., Canedo A.D., Horn F., Tamajusuku A.S., Wink M.R., Salbego C., Battastini A.M. (2006). Antiproliferative effect of quercetin in the human U138MG glioma cell line. Anticancer Drugs.

[43] Caltagirone S., Raneletti F.O., Rinelli A., Maggiano N., Colasante A., Musiani P., Aiello F.B., Piantelli M. (1997). Interaction with type II estrogen binding sites and antiproliferative activity of tamoxifen and quercetin in human non-small-cell lung cancer. Am. J. Respir. Cell Mol. Biol..

[44] Caltagirone S., Rossi C., Poggi A., Ranelletti F.O., Natali P.G., Brunetti M., Aiello F.B., Piantelli M. (2000). Flavonoids apigenin and quercetin inhibit melanoma growth and metastatic potential. Int. J. Cancer.

[45] Choi E.J., Bae S.M., Ahn W.S. (2008). Antiproliferative effects of quercetin through cell cycle arrest and apoptosis in human breast cancer MDA-MB-453 cells. Arch. Pharm. Res..

[46] Choi J.A., Kim J.Y., Lee J.Y., Kang C.M., Kwon H.J., Yoo Y.D., Kim T.W., Lee Y.S., Lee S.J. (2001). Induction of cell cycle arrest and apoptosis in human breast cancer cells by quercetin. Int. J. Oncol..

[47] Chen A.Y., Chen Y.C. (2013). A review of the dietary flavonoid, kaempferol on human health and cancer chemoprevention. Food Chem..

[48] Kim B.W., Lee E.R., Min H., Jeong H.S., Ahn J.Y., Kim J.H., Choi H.Y., Choi H., Kim E.Y., Park S.P. (2008). Sustained ERK activation is involved in the kaempferol-induced apoptosis of breast cancer cells and is more evident under 3-D culture condition. Cancer Biol. Ther..

[49] Hong J.T., Yen J.H., Wang L., Lo Y.H., Chen Z.T., Wu M.J. (2009). Regulation of heme oxygenase-1 expression and MAPK pathways in response to kaempferol and rhamnocitrin in PC12 cells. Toxicol. Appl. Pharm..

[50] Kowalski J., Samojedny A., Paul M., Pietsz G., Wilczok T. (2005). Effect of apigenin, kaempferol and resveratrol on the expression of interleukin-1β and tumor necrosis factor-alpha genes in J774.2 macrophages. Pharmacol. Rep..

[51] Lee S., Kim Y.J., Kwon S., Lee Y., Choi S.Y., Park J., Kwon H.J. (2009). Inhibitory effects of flavonoids on TNF-α-induced IL-8 gene expression in HEK 293 cells. BMB Rep..

[52] Gregory S., Kelly N.D. (2011). Quercetin. Altern. Med. Rev..

[53] Monti D., Tampucci S., Chetoni P., Burgalassi S., Saino V., Centini M., Staltari L., Anselmi C. (2011). Permeation and distribution of ferulic acid and its α-cyclodextrin complex from different formulations in hairless rat skin. AAPS PharmSciTech.

[54] Li Z., Wang M., Wang F., Gu Z., Du G., Wu J., Chen J. (2007). γ-Cyclodextrin: A review on enzymatic production and applications. Appl. Microbiol. Biotechnol..

[55] Kaur N., Puri R., Jain S.K. (2010). Drug-Cyclodextrin-Vesicles Dual Carrier Approach for Skin Targeting of Anti-acne Agent. AAPS PharmSciTech.

[56] Scalia S., Tursilli R., Iannuccelli V. (2007). Complexation of the sunscreen agent, 4-methylbenzylidene camphor with cyclodextrins: Effect on photostability and human stratum corneum penetration. J. Pharm. Biomed. Anal..

[57] Monteiro M.S., Ozzetti R.A., Vergnanini A.L., de Brito-Gitirana L., Volpato N.M., de Freitas Z.M., Ricci-Júnior E., dos Santos E.P. (2012). Evaluation of octyl p-methoxycinnamate included in liposomes and cyclodextrins in anti-solar preparations: Preparations, characterizations and in vitro penetration studies. Int. J. Nanomed..

